# Prognostic relevance of the loss of stromal CD34 positive fibroblasts in invasive lobular carcinoma of the breast

**DOI:** 10.1007/s00428-020-02835-3

**Published:** 2020-05-20

**Authors:** Christina C. Westhoff, Paul Jank, Christian O. Jacke, Ute-Susann Albert, Schokufe Ebrahimsade, Peter J. Barth, Roland Moll

**Affiliations:** 1 Institute of Pathology, Philipps University of Marburg and University Hospital Giessen and Marburg GmbH, Baldingerstrasse, 35043 Marburg, Germany; 2 Department of Gynecology and Obstetrics, Breast Center Regio, Philipps University of Marburg and University Hospital Giessen and Marburg GmbH, 35043 Marburg, Germany; 3 Present Address: Scientific Institute of Private Health Insurance, 50968 Cologne, Germany; 4 Present Address: Department of Gynecology and Obstetrics, University of Wuerzburg, 97080 Wuerzburg, Germany; 5 Present Address: Pathology Practice, Rubensstrasse 125, Berlin, 12157 Germany; 6 Gerhard Domagk Institute of Pathology, University Hospital Muenster, 48149 Muenster, Germany

**Keywords:** Invasive lobular carcinoma, Breast, CD34, Fibroblasts, Fibrocytes, Cancer-associated fibroblasts, Nodal status

## Abstract

CD34+ fibroblasts are constitutive stromal components of virtually all organs, including the mammary stroma, being involved in matrix synthesis, antigen presentation, and tumor-associated stromal remodeling. The most common subtype of invasive breast carcinoma, invasive carcinoma of no special type (IBC-NST), is known for its stromal loss of CD34+ fibroblasts while acquiring alpha smooth muscle actin-positive (α-SMA+) myofibroblasts, i.e., cancer-associated fibroblasts (CAF), whereas invasive lobular carcinoma (ILC) displays partial preservation of CD34+ fibroblasts. The aim of this study was to evaluate the prognostic relevance of stromal CD34+ fibroblasts and α-SMA+ myofibroblasts in an extended collection of ILC. A total of 133 cases of ILC, primarily resected between 1996 and 2004 at University Hospital Marburg, were examined semiquantitatively for stromal content of CD34+ fibroblasts and α-SMA+ myofibroblasts. Partial preservation of CD34+ fibroblasts in the tumor stroma of ILC was confirmed. Absence of CD34+ fibroblasts in the tumor stroma significantly correlated with the presence of α-SMA+ myofibroblasts (*p* = 0.010), positive lymph node status (*p* = 0.004), and pN stage (*p* = 0.006). Stromal loss of CD34+ fibroblasts was significantly associated with lower overall and disease-free survival rates (*p* = 0.012 and 0.013, respectively). Multivariate analysis adjusted for pT and pN stage revealed stromal loss of CD34+ fibroblasts as independent prognostic parameter (*p* = 0.05). To our knowledge, this is the first report defining prognostically relevant stromal subtypes of ILC with long-term follow-up. Future research targeting the potential diagnostic and therapeutic implications of CD34+ fibroblasts and CAF in breast cancer, especially ILC, is a promising field of interest.

## Introduction

Breast cancer is the most frequent malignant tumor in women, leading both in incidence and mortality rate [1]. Invasive lobular carcinoma (ILC) is the second most common subtype with a proportion of 5–15%, following invasive carcinoma of no special type (IBC-NST) as the most common subtype with 40–75% of cases [2–4].

CD34 is a 110 kDa highly glycosylated transmembrane protein belonging to the family of sialomucins, a group of cell surface proteins including podocalyxin, thrombomucin, and endoglycan [5]. It was originally demonstrated on hematopoietic stem cells [6], but is also expressed on vascular endothelial cells and fibroblasts in diverse organs [6, 7]. The known functions of this protein include cellular adhesion, e.g., homing of T lymphocytes in lymph nodes via l-selectin [5, 6], trafficking of hematopoietic cells, enhancing proliferation and blocking differentiation [5].

CD34+ fibroblasts are constitutive stromal components of virtually all organs and may stem from bone marrow–derived circulating fibrocytes in the blood [5, 7–9]. They may secrete numerous cytokines and are believed to regulate stromal collagen content, present antigens, and facilitate angiogenesis as well as cell migration [5, 7]. Upon activation, they are known to change their phenotype and acquire alpha smooth muscle actin (α-SMA) expression [8, 10], being involved in wound healing as myofibroblasts and pathologically representing an important player in the tumor stroma, known as cancer-associated fibroblast (CAF) [7, 8, 10]. As such, the net effect of CAF seems to be predominantly pro-tumorigenic, driving tumor cells toward invasive and metastatic properties [8, 10, 11]. In the context of anti-tumor therapy, CAFs are viewed as conferring drug resistance by impaired drug delivery and biochemical signaling [10] while on the other hand being used as tumor-targeted therapeutic vehicles by delivering chemokines, prodrugs, and oncolytic viruses, or accumulating and delivering anti-neoplastic agents [8]. CAFs have even been identified as an adverse prognostic factor in different cancer types of different origins [10], including breast cancer [12]. However, due to the consistent loss of CD34 expression, CAF do no longer represent genuine CD34+ fibroblasts. In fact, CD34+ fibroblasts are generally absent in the stroma of most carcinomas as has been demonstrated for a variety of carcinoma entities [13–20].

In the breast, Yamazaki and Eyden first described CD34+ fibroblasts in mammary stroma, with intralobular fibroblasts (i.e., directly adjacent to the epithelial cells of the terminal ductal lobular unit (TDLU)) being more numerous than interlobular fibroblasts (i.e., between TDLU) [6]. Benign breast lesions such as ductal hyperplasia, sclerosing adenosis, fibroadenoma (FA), and phyllodes tumor (PT) retain these stromal CD34+ fibroblasts, while FA and PT acquire additional α-SMA+ myofibroblasts [13]. In ductal carcinoma in situ and IBC-NST, all cases were negative for CD34+ fibroblasts, while a substantial proportion exhibited α-SMA+ myofibroblasts in the tumor stroma instead [13]. The same pattern was demonstrated in tubular carcinoma of the breast, with the constraint of a similar pattern in the central part of radial scars, underlining their close relationship [16]. However, for ILC, we reported the exceptional finding that CD34+ fibroblasts are partially preserved in the tumor stroma [21]. The biological and clinical significance of the presence of CD34+ fibroblasts in ILC and the pronounced variability in CD34+ fibroblast content within different ILC cases remained unclear until now. In the present study, we analyzed an extended collection of ILC for the presence of CD34+ fibroblasts and CAF. On the basis of clinicopathological and follow-up analyses, we here report a significant prognostic relevance of the loss of CD34+ fibroblasts in the stroma of ILC.

## Materials and methods

The archive of the Institute of Pathology, Marburg, was searched for all cases of invasive lobular carcinoma of the breast with primary surgery and date of surgery between April 1, 1996 and December 31, 2004 (*n* = 230). The study was approved by the Ethics Committee of the Medical Faculty of the Philipps University of Marburg (Az. 135/13). The corresponding clinical data were extracted, partially from two prospective cohort studies including all incident cases of malignant breast cancer in two distinct time periods from 1996 to 1997 and 2003–04. Data quality was extensively assured [22]. The clinical data and vital status were assessed until December 2018 with observation periods up to 21 years. Representative paraffin blocks from *n* = 133 tumors were available with complete information regarding nodal status at surgery. The clinicopathological characteristics of the patients involved in this study are displayed in Table 1. Most cases showed classical ILC, a few cases tubulolobular pattern, and only single cases solid, pleomorphic, or mixed patterns.

**Table 1:** Clinicopathological characteristics of the patients involved in this study

	No. of cases (% of *n*)	*n*
pT stage		133
pT1a	9 (6.77%)	
pT1b	13 (9.77%)	
pT1c	59 (44.4%)	
pT2	38 (48.6%)	
pT3	11 (8.27%)	
pT4b	3 (2.26%)	
Grading		133
G1	6 (4.51%)	
G2	101 (75.9%)	
G3	26 (19.5%)	
Lymph node positivity		133
No	89 (66.9%)	
Yes	44 (33.1%)	
pN stage		133
pN0	89 (66.9%)	
pN1mi	4 (3.01%)	
pN1a	15 (11.3%)	
pN2a	14 (10.5%)	
pN3a	10 (7.52%)	
pN3b	1 (0.75%)	
Distant metastasis until December 2018		99
With metastasis	26 (26.3%)	
Without metastasis	73 (73.7%)	
Morphological ILC subtype		133
Classical ILC	112 (84.2%)	
Solid ILC	1 (0.75%)	
Pleomorphic ILC	1 (0.75%)	
Tubulolobular ILC	18 (13.5%)	
Mixed	1 (0.75%)	

Tissues were fixed in 4% formalin solution, embedded in paraffin, cut at a thickness of 4 μm, and stained with hematoxylin and eosin (H&E) for routine purposes. Immunohistochemistry was performed using standard methods (BOND Polymer Refine Detection, Leica, Wetzlar, Germany, with 3,3′-diaminobenzidine (DAB) as chromogen). CD34 and alpha smooth muscle actin (α-SMA) were detected by the monoclonal antibodies QBEnd10 (Dako, Hamburg, Germany) and ASM-1 (Progen, Heidelberg, Germany), respectively. The immunostainings were run on an automated immunostaining apparatus (Leica BOND-MAX, Leica, Wetzlar, Germany).

The immunoreactivity of both CD34 and α-SMA in the stromal fibroblasts was graded semiquantitatively as negative (≤ 10% of the tumor area), focally positive (11–50% of the tumor area), predominantly positive (51–90% of the tumor area), and uniformly positive (> 90% of the tumor area). Percentages were assessed by two independent observers, discrepancies were discussed with a third independent observer, and consensus was reached.

Statistical analysis was performed using R and the compareGroups package [23, 24]. Differences in immunoreactivity were explored with regard to TNM stage (tumor size in mm, pT- and pN-stage and distant metastasis until December 2018) and grading and were evaluated with t-, *χ*2- or Fisher’s exact-tests where appropriate. Differences in immunoreactivity were also evaluated with respect to overall survival and disease-free survival with Kaplan-Meier curves and log-rank tests. Univariate, bivariate, and multivariate Cox regression analysis was carried out regarding vital status as a dependent variable and dichotomous evaluation of CD34 expression in stromal fibroblasts, pT stage, and pN stage as independent variables [25].

## Results

**Fig. 1:**
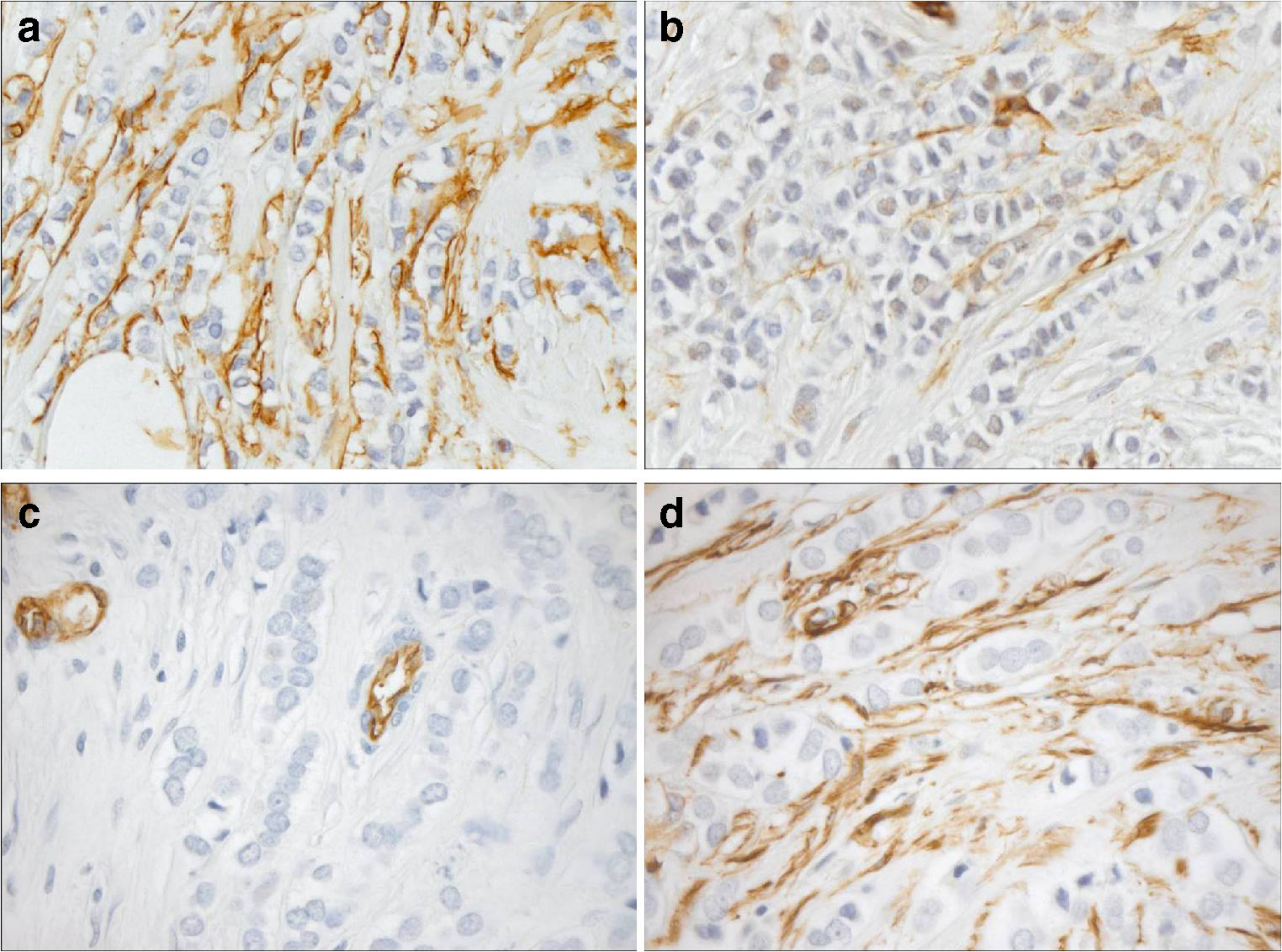
Staining patterns of fibroblasts for CD34 and αSMA in two selected cases of invasive lobular carcinoma (ILC) of the breast (**a** and **c** CD34 immunohistochemistry. **b** and **d** αSMA immunohistochemistry). **a**–**b** A case of ILC with homogenous distribution of CD34+ fibroblasts in the tumor stroma (a) and moderate density of αSMA+ myofibroblasts in the tumor stroma (b). **c**–**d** A case of ILC with loss of CD34+ fibroblasts in the tumor stroma (internal positive control of CD34+ endothelium, (c)) and homogenous distribution of strongly αSMA+ myofibroblasts in the tumor stroma (d)

Consistent with our previously published data [21], semiquantitative assessment of the proportion of CD34+ fibroblasts in the tumor stroma of ILC revealed their partial preservation, with pronounced intertumoral heterogeneity (Table 2). Figure 1 presents typical staining patterns of fibroblasts for CD34 and αSMA in two selected cases of ILC.

**Table 2:** Semiquantitative assessment of proportion of CD34+ fibroblasts in tumor stroma of invasive lobular carcinoma of the breast; continuous variables: mean (standard deviation), *p* values of the *t* test for association with clinicopathological parameters; categorical variables: absolute (relative) frequencies, *p* values of the *χ*2- or Fisher’s exact-tests for association with clinicopathological parameters

Proportion of CD34+ fibroblasts	Absent *n* = 49	Focally present *n* = 49	Predominantly present *n* = 15	Uniformly present *n* = 20	*p* overall
Tumor size, in mm	25.2 (17.5)	24.3 (18.5)	14.3 (7.59)	22.8 (22.0)	0.219
pT stage					0.615
pT1a	2 (4.08%)	4 (8.16%)	1 (6.67%)	2 (10.0%)	
pT1b	4 (8.16%)	2 (4.08%)	3 (20.0%)	4 (20.0%)	
pT1c	21 (42.9%)	22 (44.9%)	8 (53.3%)	8 (40.0%)	
pT2	16 (32.7%)	16 (32.7%)	3 (20.0%)	3 (15.0%)	
pT3	4 (8.16%)	4 (8.16%)	0 (0.00%)	3 (15.0%)	
pT4b	2 (4.08%)	1 (2.04%)	0 (0.00%)	0 (0.00%)	
Grade					0.067
G1	0 (0.00%)	2 (4.08%)	1 (6.67%)	3 (15.0%)	
G2	38 (77.6%)	41 (83.7%)	10 (66.7%)	12 (60.0%)	
G3	11 (22.4%)	6 (12.2%)	4 (26.7%)	5 (25.0%)	
Lymph node positivity					0.004
Yes	24 (49.0%)	16 (32.7%)	1 (6.67%)	3 (15.0%)	
No	25 (51.0%)	33 (67.3%)	14 (93.3%)	17 (85.0%)	
pN stage					0.006
pN0	25 (51.0%)	33 (67.3%)	14 (93.3%)	17 (85.0%)	
pN1mi	2 (4.08%)	1 (2.04%)	0 (0.00%)	1 (5.00%)	
pN1a	3 (6.12%)	11 (22.4%)	1 (6.67%)	0 (0.00%)	
pN2a	11 (22.4%)	2 (4.08%)	0 (0.00%)	1 (5.00%)	
pN3a	7 (14.3%)	2 (4.08%)	0 (0.00%)	1 (5.00%)	
pN3b	1 (2.04%)	0 (0.00%)	0 (0.00%)	0 (0.00%)	
Distant metastasis until 12/2018					0.231
With metastasis	14 (33.3%)	9 (25.7%)	0 (0.00%)	3 (23.1%)	
Without metastasis	28 (66.7%)	26 (74.3%)	9 (100%)	10 (76.9%)	
Proportion of α SMA myofibroblasts					0.010
Absent	12 (24.5%)	14 (28.6%)	6 (40.0%)	13 (65.0%)	
Focally present	12 (24.5%)	14 (28.6%)	7 (46.7%)	5 (25.0%)	
Predominantly present	15 (30.6%)	16 (32.7%)	2 (13.3%)	2 (10.0%)	
Uniformly present	10 (20.4%)	5 (10.2%)	0 (0.00%)	0 (0.00%)	
Proportion of α SMA myofibroblasts, dichotomized					0.010
Absent	12 (24.5%)	14 (28.6%)	6 (40.0%)	13 (65.0%)	
Present	37 (75.5%)	35 (71.4%)	9 (60.0%)	7 (35.0%)	
Morphological ILC subtype					0.982
Classical ILC	39 (79.6)	42 (85.7%)	13 (86.7%)	18 (90.0%)	
Solid ILC	1 (2.04%)	0 (0.00%)	0 (0.00%)	0 (0.00%)	
Pleomorphic ILC	1 (2.04%)	0 (0.00%)	0 (0.00%)	0 (0.00%)	
Tubulolobular ILC	7 (14.3%)	7 (14.3%)	2 (13.3%)	2 (10.0%)	
Mixed	1 (2.04%)	0 (0.00%)	0 (0.00%)	0 (0.00%)

**Fig. 2:**
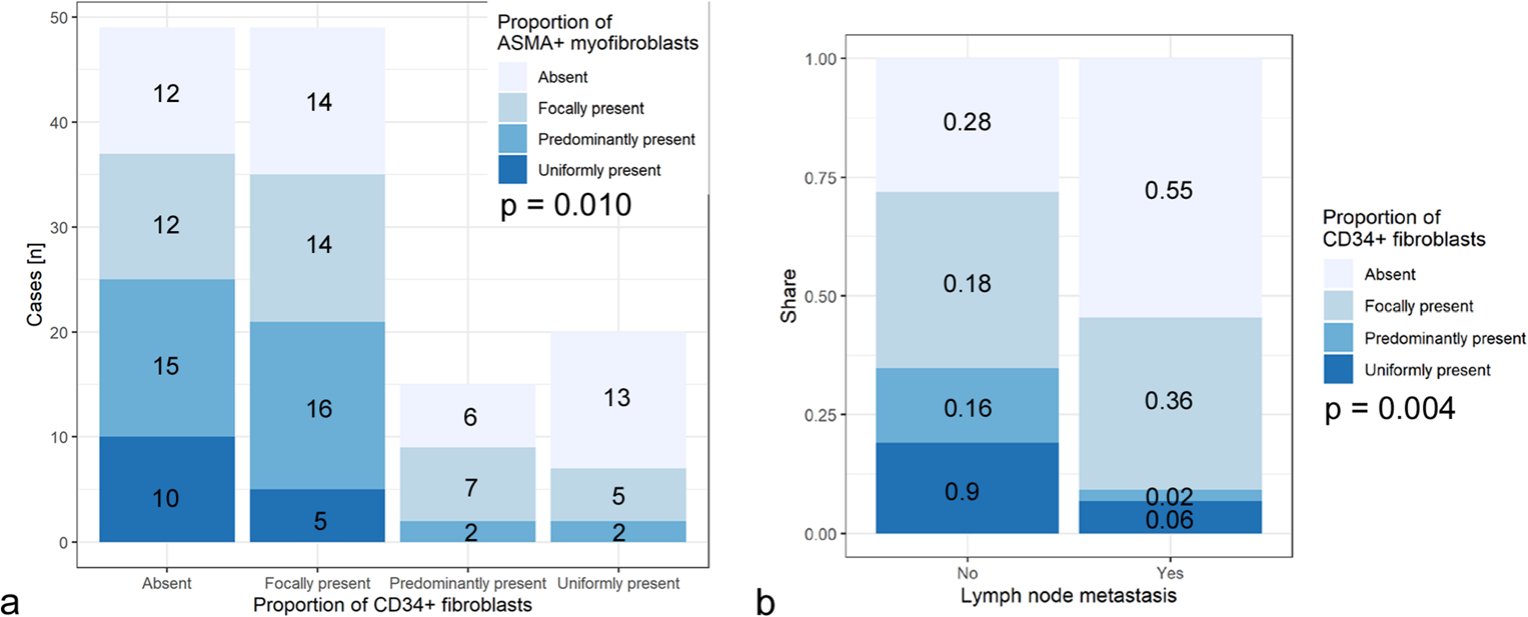
Stacked bar plots representing the semiquantitatively assessed proportion of CD34+ stromal fibroblasts with respect to the proportion of αSMA (ASMA) + stromal myofibroblasts (**a**, absolute number of cases) and the status of lymph node metastasis (**b**, proportion of cases)

Semiquantitative assessment of the proportion of CD34+ fibroblasts in the tumor stroma of invasive lobular carcinoma of the breast showed no statistically significant difference for tumor size, pT stage, grading, distant metastasis until December 2018, or tumor type (Table 2). However, the absence of CD34+ fibroblasts in the tumor stroma statistically significantly correlated with the presence of αSMA+ myofibroblasts in the tumor stroma, both in the semiquantitative and the dichotomized analyses (Table 2, Fig. 2a). Also, the absence of CD34+ stromal fibroblasts is strongly correlated with the positive lymph node status and pN stage, respectively (Table 2, Fig. 2b).

**Fig. 3:**
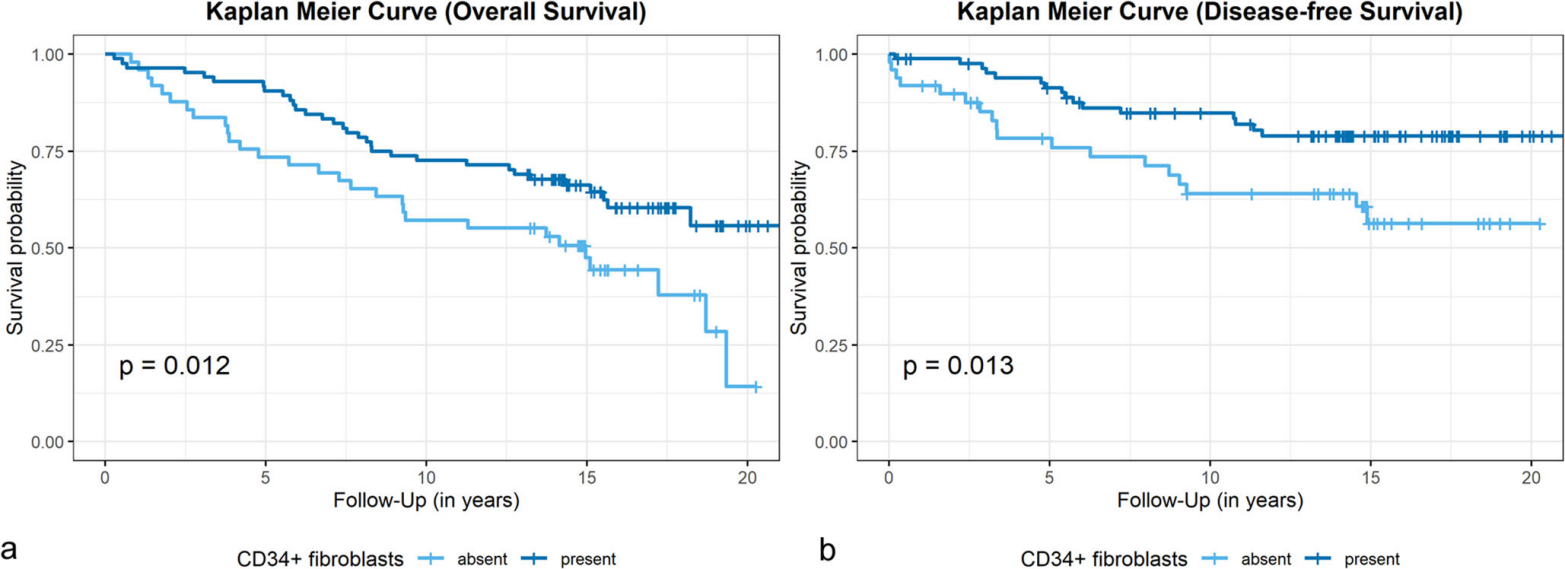
Kaplan-Meier curves for overall survival (**a**) and disease-free survival (**b**) with respect to the presence (“Present”) or absence (“Absent”) of stromal CD34+ fibroblasts (positive cases, *n* = 84; negative cases, *n* = 49), *p* values of the log-rank tests

We performed Kaplan-Meier analyses for assessment of prognostic relevance based on the dichotomized analysis. Stromal loss of CD34+ fibroblasts was significantly associated with lower overall overall survival (OS) rates (5-year overall survival CD34−, 73%; CD34+, 90%; *p* = 0.012), (Fig. 3a) and lower disease-free survival (DFS) rates (5-year disease-free survival CD34−, 75%; CD34+, 91%; *p* = 0.013) (Fig. 3b). The presence of stromal ASMA+ myofibroblasts was not significantly associated with OS or DFS (data not shown).

Higher pT and pN stage and distant metastasis until December 2018 were significantly associated with lower OS (*p* < 0.001), indicating a representative study population (data not shown).

**Fig. 4:**
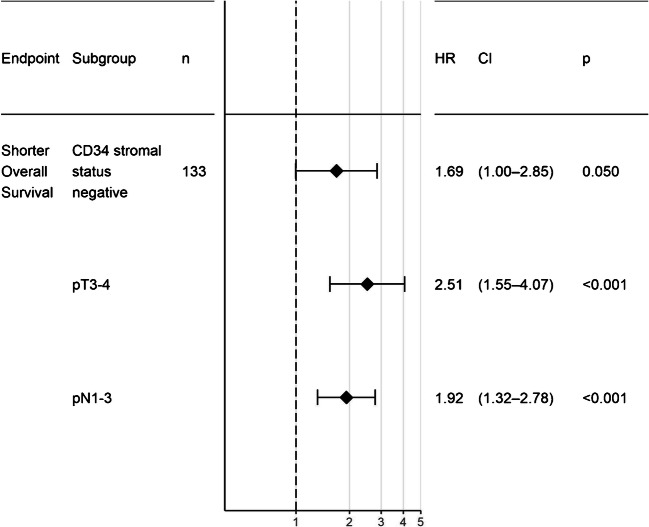
Forest plot for the multivariate analysis plotting the Cox regression hazard ratio (HR), 95% confidence interval (CI), and *p* values with respect to overall survival for loss of CD34+ fibroblasts (“CD34 stromal status negative”) vs. preservation of CD34+ fibroblasts, pT stages 3 and 4 (“pT3–-4”) vs. pT stages 1 and 2, and positive nodal status (“pN1–3”) vs. negative nodal status

Multivariate analysis of stromal loss of CD34+ fibroblasts vs. pT and pN stage revealed that a negative stromal status for CD34 is an independent prognostic parameter (*p* = 0.05, Fig. 4).

## Discussion

CD34+ fibroblasts are constitutive stroma components of most tissues, including the female breast [6, 7]. IBC-NST of the breast consistently lacks CD34+ fibroblasts in the tumor stroma [13], while invasive lobular carcinomas display a more complex phenotype with partial preservation of CD34+ stromal fibroblasts and pronounced intertumoral heterogeneity [21]. Previous, smaller studies were inconsistent with respect to a potential correlation between the loss of CD34+ fibroblasts and positive lymph node status [26, 27]. This larger study population with long-term follow-up emphasizes that the absence of CD34+ fibroblasts in the tumor stroma of a subset of ILC is associated with the presence of αSMA+ myofibroblasts and therefore indicates a phenotypic switch to cancer-associated fibroblasts (CAFs) for this subgroup. This subset of ILC with loss of CD34+ fibroblasts resembles the stromal characteristics of IBC-NST [13], whereas 26% of the studied cases demonstrate a predominantly or uniformly positive tumor stroma for CD34+ fibroblasts, pointing to the existence of distinct stromal subtypes for ILC. So far, only one other tumor entity is known for this peculiar pattern: diffuse gastric carcinoma [15], sharing further similarities with ILC, e.g., discohesive growth pattern and loss of E-Cadherin expression [28].

While there was no statistically significant difference for tumor size, pT stage, grading, or distant metastasis until December 2018 with respect to the proportion of CD34+ fibroblasts in the tumor stroma of ILC, the absence of CD34+ stromal fibroblasts strongly correlated with positive lymph node status and the respective pN stage, being in line with earlier findings [26] of our group. In the present, extended study, we additionally demonstrate that stromal loss of CD34+ fibroblasts is significantly associated with lower overall and disease-free survival rates. Multivariate analysis indicates the stromal loss of CD34+ fibroblasts being an independent prognostic factor.

According to the comprehensive review of Christgen et al. [4], only few of the cited studies demonstrated an association of ILC with higher pN stage in comparison with IBC-NST, while Mamtani et al. state lobular histology being an independent predictor of nodal micrometastasis [29]. However, none of these studies differentiated ILC according to their stromal characteristics, e.g., with respect to the presence of CD34+ fibroblasts.

Nakagawa et al. studied grade- and age-matched luminal-like ILC and IBC-NST with respect to the tumor microenvironment [30], characterized by localization of CD34, αSMA, CD31, Vashibin-1, and nestin.

According to the authors, all tumors were CD34 negative, whereas the number of αSMA+ CAF, microvessel density, and vasohibin-1/CD31 ratio was significantly higher in ILC, but nestin immunoreactivity was lower in ILC. The authors conclude that proliferation of CAF and endothelial cells in ILC is more pronounced, but the newly formed microvessels are less mature. Astonishingly, among 145 ILC cases, there was no single one with at least partial preservation of CD34+ fibroblasts in the tumor stroma, contrary to our experience. Also, we have not observed a qualitative difference between IBC-NST and ILC regarding the presence of αSMA+ CAF [13, 21].

Although CAFs are viewed as key regulators of the tumor microenvironment as well as a potential therapeutical target in breast cancer [11, 12], only few studies exist on potential differences with respect to ILC and IBC-NST [31, 32].

Park et al. investigated numerous CAF-related proteins with respect to tumoral and stromal localization as well as tumor type (IBC-NST vs. ILC) [31]. They found statistically relevant differential expression for localization and tumor type but no prognostic effect for ILC alone. When analyzing all tumor types, they found tumoral and stromal CAF-related proteins associated with worse DFS or OS, overall without clear preference for either tumor type.

Schoppmann et al. evaluated podoplanin expression in CAF in invasive breast cancer without a statistically relevant difference between IBC-NST and ILC. In multivariate analysis, podoplanin expression in CAF was an independent prognostic factor for worse DFS and OS [32]. Interestingly, cases with podoplanin expressing CAF were associated with negative lymph node status.

This contrasts our finding of loss of CD34+ fibroblasts (and presence of αSMA+ CAF) correlating with positive lymph node status, possibly being due to the use of different CAF-markers.

Few studies evaluated markers of CAF in breast cancer with respect to lymph node metastasis. One systematic review with meta-analysis and own cases found expression of matrix metalloproteinase 13 (MMP13) in CAF significantly associated with positive axillary nodal status, whereas caveolin-1 (CAV) expression in CAF was significantly associated with negative axillary nodal status [33]. However, these outcomes were valid in the meta-analysis only. In most papers evaluated, IBC-NST predominated by far, and most papers showed no significant difference between IBC-NST and ILC.

Therapeutic approaches target CAF in different ways, e.g., targeting fibroblast activation protein (FAP) by T cells or vaccination, aiming at CAF-derived immunosuppressive molecules like TGF-β or using hedgehog or tyrosin kinase inhibitors [8, 10–12]. Our results could add valuable information as to the potential individual benefit for each patient from potentially harmful therapeutic agents, since patients without relevant stromal changes, i.e., without CAF, might not benefit from these therapeutic strategies. This is especially true for ILC, since clinical trials and histopathological studies often do not differentiate between different types of breast cancer in spite this being the second most common histological subtype of breast cancer.

Other papers analyzing the tumor microenvironment of ILC refer to PD-L1 expression [34], with a subset of ILC expressing PD-L1 on tumor cells and harboring PD-L1+ tumor-infiltrating lymphocytes (TIL), but there was no association between higher TIL and PD-L1 labeling with ER status as seen in IBC-NST. Davies et al. linked PD-L1 expression to CAF (or in their nomenclature mesenchymal stromal cells (MSC)) and report expression and secretion of PD-L1 and PD-L2 by MSC being regulated by interferon-γ and tumor necrosis factor-α [35]. Since ILC responds less well to chemotherapy than IBC-NST, targeted immunotherapy by antibodies against PD-L1 might constitute a promising treatment option for at least a subset of ILC [34]. It would be interesting to further exploit the potential influence of CAF in this context.

To our knowledge, this is the first report defining prognostically relevant stromal subtypes of ILC. In contrast to the more common stromal subtype with (partially or uniformly) present CD34+ fibroblasts, the stromal subtype with absent CD34+ fibroblasts displays stromal features equivalent to that typically seen in IBC-NST, with a switch to αSMA+ CAF [13]. The presence of αSMA+ CAF is associated with worse prognosis, in breast cancer and other tumor types [10, 12], the presence of CAF is also associated with a higher rate of metastasis [8, 11, 12]. Further studies are required to estimate whether the analysis of CD34+ fibroblasts should be included in the diagnostic panel of ILC.

## Conclusion

Analyzing a large cohort of ILC with long-term follow-up information confirmed partial preservation of CD34+ stromal fibroblasts in contrast to IBC-NST. Stromal loss of CD34+ fibroblasts in a subset of ILC correlated with the appearance of αSMA+ myofibroblasts (CAF), similarly to IBC-NST in general. Stromal loss of CD34+ fibroblasts correlated with the presence of lymph node metastases and, in addition, with worse overall and disease-free survival. To our knowledge, this is the first report defining prognostically relevant stromal subtypes of ILC. Multivariate analysis of stromal loss of CD34+ fibroblasts vs. pT and pN stage disclosed negative stromal status for CD34 being an independent prognostic parameter. Future research targeting the potential diagnostic and therapeutic implications of CD34+ fibroblasts and CAF in breast cancer and especially ILC is a promising field of interest.
